# MCR-1 Inhibition with Peptide-Conjugated Phosphorodiamidate Morpholino Oligomers Restores Sensitivity to Polymyxin in *Escherichia coli*

**DOI:** 10.1128/mBio.01315-17

**Published:** 2017-11-07

**Authors:** Seth M. Daly, Carolyn R. Sturge, Christina F. Felder-Scott, Bruce L. Geller, David E. Greenberg

**Affiliations:** a Department of Internal Medicine, UT Southwestern Medical Center, Dallas, Texas, USA; b Department of Microbiology, Oregon State University, Corvallis, Oregon, USA; c Department of Microbiology, UT Southwestern Medical Center, Dallas, Texas, USA; Louis Stokes Veterans Affairs Medical Center

**Keywords:** PPMO, antisense, colistin, *mcr-1*, polymyxins

## Abstract

In late 2015, the first example of a transferrable polymyxin resistance mechanism in Gram-negative pathogens, MCR-1, was reported. Since that report, MCR-1 has been described to occur in many Gram-negative pathogens, and the mechanism of MCR-1-mediated resistance was rapidly determined: an ethanolamine is attached to lipid A phosphate groups, rendering the membrane more electropositive and repelling positively charged polymyxins. Acquisition of MCR-1 is clinically significant because polymyxins are frequently last-line antibiotics used to treat extensively resistant organisms, so acquisition of this mechanism might lead to pan-resistant strains. Therefore, the ability to inhibit MCR-1 and restore polymyxin sensitivity would be an important scientific advancement. Peptide-conjugated phosphorodiamidate morpholino oligomers (PPMOs) are antisense molecules that were designed to target mRNA, preventing translation. Peptide conjugation enhances cellular entry, but they are positively charged, so we tested our lead antibacterial PPMOs by targeting an essential *Escherichia coli* gene, *acpP*, and demonstrated that they were still effective in *mcr*-*1*-positive *E. coli* strains. We then designed and synthesized two PPMOs targeted to *mcr-1* mRNA. Five clinical *mcr-1*-positive *E. coli* strains were resensitized to polymyxins by MCR-1 inhibition, reducing MICs 2- to 16-fold. Finally, therapeutic dosing of BALB/c mice with MCR-1 PPMO combined with colistin in a sepsis model reduced morbidity and bacterial burden in the spleen at 24 h and offered a survival advantage out to 5 days. This is the first example of a way to modulate colistin resistance with an antisense approach and may be a viable strategy to combat this globally emerging antibiotic resistance threat.

## IMPORTANCE

Polymyxin use has been increasing as a last line of defense against Gram-negative pathogens with high-level resistance mechanisms, such as carbapenemases. The recently described MCR-1 is a plasmid-mediated mechanism of resistance to polymyxins. MCR-1 is currently found in Gram-negative organisms already possessing high-level resistance mechanisms, leaving clinicians few or no antibacterial options for infections caused by these strains. This study utilizes antisense molecules that target mRNA, preventing protein translation. Herein we describe antisense molecules that can be directly antibacterial because they target genes essential to bacterial growth or blockade of MCR-1, restoring polymyxin sensitivity. We also demonstrate that MCR-1 antisense molecules restore the efficacies of polymyxins in mouse models of *E. coli* septicemia. Considering all things together, we demonstrate that antisense molecules may be effective therapeutics either alone when they target an essential gene or combined with antibiotics when they target specific resistance mechanisms, such as those seen with MCR-1.

## INTRODUCTION

The rise in antibiotic resistance in Gram-negative bacteria, especially carbapenem resistance, coupled with the dearth of new antibiotics has required the use of the older polymyxins, colistin (polymyxin E) and polymyxin B, which have worrisome toxicities (1). Polymyxin is used quite extensively in the agricultural domain, with an estimated 1.2 × 10^7^ kg having been used in China in 2015 (2). In late 2015, the first plasmid-mediated mechanism of resistance to the polymyxins, *mcr-1*, was discovered in livestock, food meat, and humans in China (2). This led to a global explosion in the number of publications describing the presence of *mcr-1* in a large number of Gram-negative pathogens isolated from a variety of sources and countries and the mechanistic description of MCR-1 (we obtained 305 results for the PubMed search “mcr-1” since November 2015). The gene encodes a phosphoethanolamine transferase, which alters the charge of lipid A from electronegative to electropositive; since the polymyxins are cationic, they are thereafter prevented from interacting with the membrane and cannot exert their antimicrobial activity (3–5).

The loss of these last-line-of-defense antibiotics requires the development of novel and effective therapeutics. Phosphorodiamidate morpholino oligomers (PMOs) are short synthetic antisense molecules that are targeted to mRNAs to inhibit protein translation by steric blockade. They are conjugated to short cationic peptides (PPMOs), termed cell-penetrating peptides, to aid in cellular entry. PPMOs have demonstrated success against bacteria when targeted to (i) essential genes, killing bacteria like antibiotics (6–9); (ii) resistance genes, rendering bacteria sensitive to antibiotics (10, 11); and (iii) virulence genes, preventing pathogenesis (unpublished observations). We sought to demonstrate that PPMO blockade of MCR-1 production would restore colistin efficacy in *Escherichia coli*; however, because the PPMOs are conjugated to positively charged peptides, we first wanted to determine whether PPMOs would be effective against an *mcr-1*-positive organism.

## RESULTS

### PPMOs targeted to essential genes inhibit *mcr-1*-positive *E. coli*.

**FIG 1:**
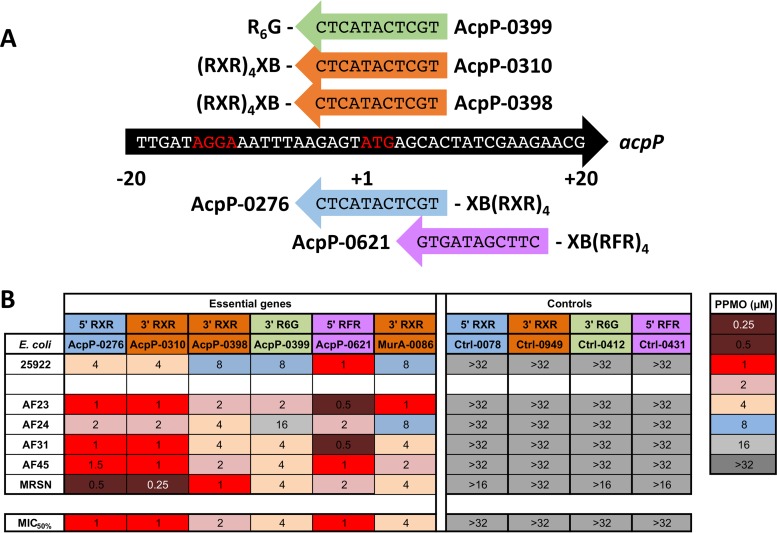
Positively charged PPMOs are still effective against *mcr-1*-positive *E. coli*. (A) PPMOs targeted to the *acpP* gene sequence of *E. coli* MG1655 are displayed with their designation numbers and conjugated peptides. The locations of the peptides indicate the 5′ versus 3′ attachment (X indicates 6-hexanoic acid, and B indicates beta-alanine). The Shine-Dalgarno and ATG start site are indicated in red. (B) The MIC_50_ values for the five *mcr-1*-positive strains are presented compared to that for the standard, ATCC 25922. PPMOs targeting the essential genes for acyl carrier protein (*acpP*) and UDP-*N*-acetylglucosamine 1-carboxyvinyltransferase (*murA*) are depicted to the left, and appropriate controls are to the right. The color coding of the PPMOs refers to the four different peptide conjugates for comparison. MRSN, MRSN 388634.

Since PMOs are conjugated to positively charged peptides and electrostatic repulsion is the mechanism of polymyxin resistance, we first sought to determine whether PPMOs would be effective against the positively charged membrane of *mcr-1*-positive strains (colistin MICs of 4 to 8 μg/ml). The *E. coli* strains AF23 to -45 are clinical isolates that possess other resistance markers, such as TEM-1 and CTX-M-55 (12); MRSN 388634 was the first clinical *E. coli* isolate in the United States and also harbors multiple resistance markers (13). To complete this objective, we utilized PPMOs targeted to the essential gene *acpP* (Fig. 1A), which we have previously shown to inhibit growth in *E. coli* (6–8). We selected AcpP PPMOs which target different regions of the *acpP* start site, PPMOs which had different sites of peptide attachment (5′ versus 3′), and different peptides to optimize the PPMO chemistry (Fig. 1A). The MIC values ranged from 0.25 to 16 μM for the AcpP PPMOs, while the relevant control PPMOs (Ctrl PPMOs) were not active at up to 32 μM (Fig. 1B). The MIC_50_ values, or the concentrations required to inhibit 50% of *mcr-1*-positive strains, ranged from 1 to 4 μM (Fig. 1B). When we compared AcpP PPMOs, all three PPMOs with different cell-penetrating peptides [(R)_6_G, (RXR)_4_XB, and (RFR)_4_XB] were efficacious, and neither the site of attachment (AcpP-310’s site is 3′ versus AcpP-0276’s, which is 5′) nor the chemistry for synthesis (AcpP-310, hydrogen, versus AcpP-398, triethylene glycol) affected the MIC (see Table S1 in the supplemental material). Surprisingly, for most of the strains and PPMO combinations, the PPMO worked better in the *mcr-1*-positive strain than in the standard CLSI *E. coli* reference strain (ATCC 25922) (Fig. 1B) and its MIC was comparable to MICs for other *mcr-1*-negative *E. coli* strains (unpublished observations). Finally, to demonstrate that this inhibition is not specific to *acpP*, we included a PPMO (murA-0086) targeted to the essential gene for UDP-*N*-acetylglucosamine 1-carboxyvinyltransferase (*murA*). The murA-0086 PPMO was also effective in *mcr-1*-positive strains, and these strains were more sensitive than the *E. coli* standard strain (Fig. 1B). These results demonstrate that *mcr-1*-positive *E. coli* strains, which have positively charged membranes, are still inhibited by PPMOs and that all of the conjugation peptides, sites, and chemistries tested are effective.

10.1128/mBio.01315-17.3 TABLE S1  PPMOs information. Acyl carrier protein (*acpP*), UDP-*N*-acetylglucosamine 1-carboxyvinyltransferase (*murA*), *mcr-1*, and control (Ctrl) PPMO information is depicted. The A of ATG designates position 1 for the locations on genes; the 5′ direction begins with position −1. Noncanonical peptides are X-6-aminohexanoic acid and B-beta-alanine. TEG, triethylene glycol. Download TABLE S1, TIF file, 0.5 MB. Copyright © 2017 Daly et al. 2017 Daly et al. This content is distributed under the terms of the Creative Commons Attribution 4.0 International license.

### MCR-1 PPMOs restore *E. coli* sensitivity to polymyxins.

**FIG 2:**
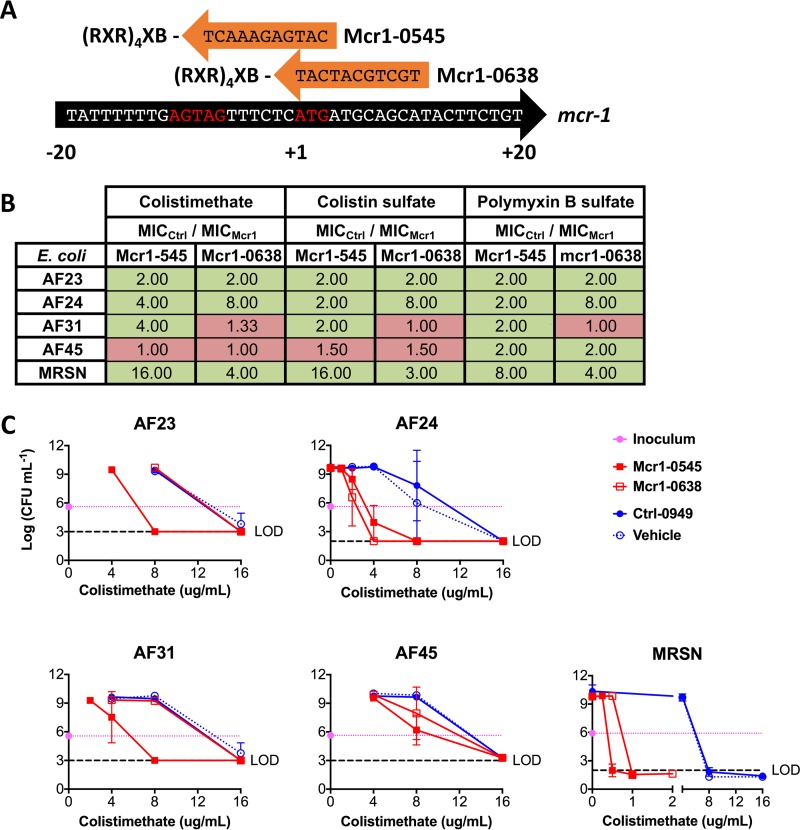
PPMOs targeting *mcr1* restore *E. coli* polymyxin sensitivity. (A) 3′-end-conjugated PPMOs targeted to the *mcr-1* sequence of *E. coli* SHP45 are displayed as in Fig. 1 (X indicates 6-hexanoic acid, and B indicates beta-alanine). (B) Fold enhancement of the MICs of the three most common formulations of polymyxin, i.e., colistimethate and colistin sulfate (polymyxin E) and polymyxin B sulfate, by the two MCR-1 PPMOs at 16 μM. Fold enhancement of the MIC is represented as MIC_Ctrl PPMO_/MIC_MCR-1 PPMO_. Green boxes represent an enhancement (≥2), and red boxes represent no enhancement (≤2). (C) CFU determination of the *mcr-1*-positive strains following MIC measurement. Pink lines indicate the inoculum, and dashed black lines indicate the CFU limit of detection (LOD).

The (RXR)_4_XB peptide has proven efficacious in many Gram-negative genera (7, 9–11, 14), so we designed two MCR-1 PPMOs (Mcr1-0545 and Mcr1-0638) (Fig. 2A) based on the first-described *mcr-1*-positive *E. coli* strain, SHP45 (2). The standard MIC assay was then conducted with a fixed 16 μM concentration of the MCR-1 PPMOs, Ctrl-0949, or vehicle (H_2_O) combined with the three most common polymyxin forms. The MCR-1 and Ctrl PPMOs were included in the assays alone at 16 μM, and antibacterial activity was never detected (data not shown); a growth curve was also generated for MRSN 388634, and no change in growth rate was detected, although minor alterations in the shapes of late-log and stationary-phase curves were noted (Fig. S1). The activity of each MCR-1 PPMO is calculated as the MIC of colistin with the Ctrl PPMO divided by the MIC of colistin with an MCR-1 PPMO. For most combinations, MCR-1 PPMOs decreased the MIC of colistin (Fig. 2B, green) from 2- to 16-fold. The Mcr1-0545 PPMO was more effective than Mcr1-0638. To further demonstrate the activities of the MCR-1 PPMOs, modified minimum bactericidal concentration (MBC) assays were conducted, since colistin is a bactericidal antibiotic. The trends observed in Fig. 2B were recapitulated, with less colistin being required to reach the limit of detection (Fig. 2C). These data demonstrate that (i) inhibition of MCR-1 with PPMOs leads to reduced MIC and MBC values, (ii) AF24 and MRSN 388634 are the most sensitive strains, and (iii) AF45 is the most resistant to PPMO resensitization of polymyxins.

10.1128/mBio.01315-17.1 FIG S1  MCR-1 PPMOs do not affect *E. coli*’s growth rate *in vitro*. Growth curves (optical density at 600 nm) of *E. coli* MRSN 388634 grown for 18 h in MHII with 16 μM PPMOs or water (vehicle). Download FIG S1, TIF file, 0.2 MB. Copyright © 2017 Daly et al. 2017 Daly et al. This content is distributed under the terms of the Creative Commons Attribution 4.0 International license.

### Combination therapy reduces burden and is protective *in vivo*.

**FIG 3:**
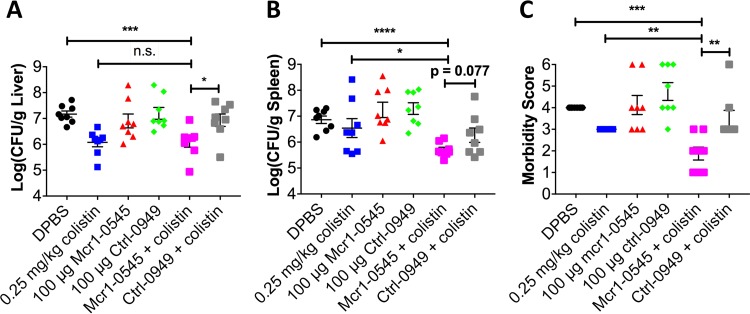
PPMOs rescue colistin activity in a mouse model of acute septicemia. Female BALB/c mice (7 to 8 weeks old) were infected via intraperitoneal injection with 8e4 CFU of MRSN 388634. At 2 and 6 h postinfection, 0.25 mg/kg colistin sulfate, 100 μg PPMO (~5 mg/kg), or the combination was administered via the i.p. route. Mice were euthanized at 24 h, and organ burden was determined. Numbers of CFU per gram of tissue of the liver (A) and spleen (B) are shown. (C) Morbidity was assessed immediately prior to euthanasia and scored as a maxima of 6 (moribund) and a nadir of 0. Mice that succumbed prior to euthanasia are depicted with the maxima score of 6. The data represent the means and standard errors of the means (SEM) of results from two replicate experiments (*n =* 4) (*n =* 8 per group total). n.s., not significant; *, *P* < 0.05; **, *P* < 0.01; ***, *P* < 0.001; ****, *P* < 0.0001 by Student’s *t* test, with the Mann-Whitney test used for morbidity.

The most potent PPMO (Mcr1-0545) was tested in a mouse model of septicemia (10). Colistin (0.25 mg/kg of body weight) and either an MCR-1 PPMO (5 mg/kg) or the Ctrl PPMO (5 mg/kg) were administered alone or in combination at 2 and 6 h postinfection, and bacterial burden was assessed at 24 h (Fig. 3). Neither the MCR-1 nor the Ctrl PPMO alone had an effect on burden or morbidity at 24 h (Fig. 3). Colistin alone resulted in a significant reduction of the burden in the liver and morbidity (Fig. 3A and C). The combination of colistin with an MCR-1 PPMO resulted in a significant reduction in the numbers of CFU in both the liver and the spleen compared to those in the Dulbecco’s phosphate-buffered saline (DPBS) control (Fig. 3A and B) and was statistically significant compared to values for colistin alone in the spleen (Fig. 3B). The liver burden and morbidity were significantly less in mice treated with a combination of an MCR-1 PPMO and colistin than in mice treated with the Ctrl PPMO and colistin (Fig. 3A and C) and approached significance in the spleen burden (*P* = 0.077) (Fig. 3B). Although the decrease in the number of CFU was modest (~1 log), the decrease in morbidity strongly corroborated the protective effects of the combination therapy compared to those of the monotherapies (Fig. 3C).

**FIG 4:**
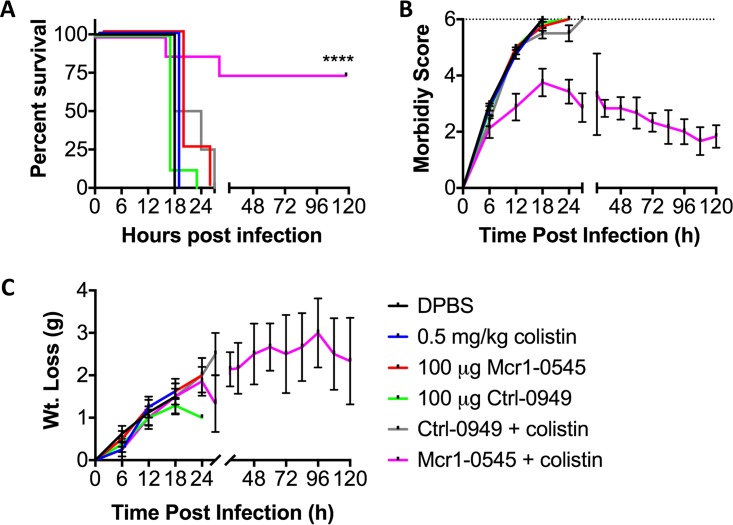
PPMOs combined with colistin protect mice from a lethal dose of *mcr-1*-positive *E. coli*. Female BALB/c mice (7 to 8 weeks old) were infected via intraperitoneal injection with 1e6 CFU of MRSN 388634. At 2 and 6 h postinfection 0.5 mg/kg colistin sulfate, 100 μg PPMO (~5 mg/kg), or the combination was administered via the i.p. route. Mice were monitored for morbidity, with an assessment scored at a maxima of 6 (moribund; dotted line [b]) and a nadir of 0. The data represent the means and SEM from two replicate experiments (*n =* 4) (*n =* 8 per group total). (A to C) Survival curve (A), morbidity scoring (B), and weight loss measurement (C) over the course of 5 days. ****, *P* < 0.0001 by the Mantel-Cox log-rank test.

The combination therapy was further tested in a mouse survival model. All mice that were dosed with a single agent succumbed to infection by 30 h, with the majority being moribund and euthanized by 18 h (Fig. 4A). However, 75% (*n* = 6/8) of the mice treated with a combination of an MCR-1 PPMO and colistin survived to the 5-day endpoint of the experiment (Fig. 4A). Increased morbidity and weight loss correlated with survival (Fig. 4B and C) in the mice treated with monotherapy or the combination therapy of the Ctrl PPMO and colistin. The morbidity of mice treated with the combination of an MCR-1 PPMO and colistin increased initially, but the mice began to recover by 24 h (Fig. 4B), although these animals continued to lose weight over the course of the experiment (Fig. 4C). These animal data demonstrate the rescue of colistin efficacy *in vivo*, with a reduction in the number of CFU at early time points and enhanced survival at 5 days postinfection.

## DISCUSSION

Given that PPMOs are highly cationic [there are eight arginines on (RXR)_4_XB], we were unsure whether they would be active in *mcr-1*-positive *E. coli* strains. We were pleased to find that PPMOs targeting both essential and nonessential genes demonstrated efficacy both *in vitro* and *in vivo*. This finding is in line with a recent report demonstrating that *mcr-1*-positive organisms are not resistant to cationic antimicrobial peptides (15). These findings are paradoxical and reflect the lack of knowledge and the complexity of the known mechanisms of cell-penetrating peptides and cationic antimicrobial peptides (16). Interestingly, PPMOs targeted to essential genes were more effective against *mcr-1*-positive strains than against the clinical *E. coli* testing standard. This is surprising, because phosphoethanolamine conjugation of lipid A is thought to reduce electrostatic repulsion in the membrane, thereby increasing packing and stability (17, 18).

The MCR-1 PPMOs sensitized *mcr-1*-positive strains of *E. coli* to three major formulations of polymyxins, which resulted in 2- to 16-fold decreases in the MICs. However, it was not surprising to discover that two other strains had no sensitization to certain polymyxins with MCR-1 PPMO treatment (Fig. 2B, red). Gram-negative organisms, including *E. coli*, possess other chromosomal enzymes that can increase the membrane’s positive charge (and therefore polymyxin resistance) by attachment of arabinose and/or phosphoethanolamine (19). Mutations in the regulators of these intrinsic mechanisms may explain the lack of activity of MCR-1 PPMOs, a potential limitation and a hypothesis that we are investigating.

We demonstrated multiple peptide attachments, including (RXR)_4_XB, that retained activity in these strains. This is important because the only data thus far on resistance to PPMOs point toward a transporter, *sbmA*, which is peptide specific (20, 21). Thus, one possible way to circumvent resistance is to utilize the same oligonucleotide linked to a novel peptide motif. In addition, the (RXR)_4_XB peptide has been shown to be effective in multiple Gram-negative genera (7, 9–11, 14, 22), and one report suggests that this motif does not rely on *sbmA* (21). Therefore, this strategy of modulating antibiotic resistance with PPMOs such as *mcr-1* should be feasible, regardless of the genera that harbor this transmissible element.

Since the description of the *mcr-1* gene (mcr-*1.1*), *mcr-1.2* through *mcr-1.8*, *mcr-2*, *mcr-3*, and *mcr-4* have been described (Fig. S2) (2, 23–29). Fortuitously, Mcr1-0545 is targeted to the region from positions −8 to +3 of *mcr-1*, which is 100% conserved in *mcr-1.x* variants (Fig. S2A), suggesting that Mcr1-0545 would be effective against these variants. Mcr1-0638 is conserved in six of the eight *mcr-1.x* strains. We have designed and synthesized *mcr-2* PPMOs and will assess their efficacy in future studies, in addition to testing PPMOs against *mcr-3* and *mcr-4*. One potential benefit of this technology is the ability to design and produce PPMOs rapidly as new single nucleotide polymorphism (SNP) (e.g., *mcr*-*1.8*) or homolog (e.g., *mcr*-*4*) variants arise. Given that the underlying chemistry is unchanged, this approach poses unique regulatory challenges that will have to be addressed. Although *mcr*-positive *Pseudomonas* and *Acinetobacter* isolates have not yet been detected, a recent study suggests that conjugative transfer is possible (30). PPMOs targeted to essential genes have demonstrated efficacy in these genera (9, 14), suggesting that an MCR-1 PPMO would be effective in these genera as well. Since *mcr-1*, *-2*, *-3*, and *-4* are not conserved, one can envision a cocktail of PPMOs that target all four variants at the same time, and future studies will address this approach.

10.1128/mBio.01315-17.2 FIG S2  Alignment of the MCR-1 PPMOs with the identified *mcr* gene variants. (A) PPMO sequences (complement) are aligned with the *mcr-1.x* gene variants (top) and *mcr-1* through *mcr-4* gene variants (bottom). Sequence homology is denoted with an asterisk, and nucleotide changes are indicated in red. (B) Citations for the original identification of each variant and the accession numbers used to build the alignments are provided. Download FIG S2, DOCX file, 0.01 MB. Copyright © 2017 Daly et al. 2017 Daly et al. This content is distributed under the terms of the Creative Commons Attribution 4.0 International license.

To our knowledge, this is the first report of a specific therapeutic that can target MCR-1 and have the potential to inhibit multiple variants and genera. Additionally, we demonstrate that essential-gene-targeted PPMOs might be effective therapeutics for treatment of strains that may harbor this resistance mechanism. Future studies will investigate activity in a broader number of *mcr-*positive isolates (*mcr-1.x* and *mcr-2* through *mcr-4*) in many genera and assess the efficacy of an MCR-1 PPMO “cocktail.”

## MATERIALS AND METHODS

### Design and synthesis of PPMOs.

PPMOs were designed by our lab and synthesized by Sarepta Therapeutics (Boston, MA) as described previously (11, 31). Briefly, we used a custom Web tool to design 11-mer PPMOs targeted near the start ATG codons of genes (https://qbrc2.swmed.edu/Greenberg/oligonucleotide5.cgi). This tool identifies PPMOs with the lowest number of mismatches between sequenced strains, in this case *E. coli* (taxon ID, 562). The PPMOs are synthesized and conjugated to peptides by Sarepta Therapeutics and delivered as lyophilizates, which are solubilized in water for use. PPMO sequence, peptide conjugation, and conjugation chemistry are listed in Table S1 in the supplemental material.

### Antibiotics and bacterial isolates.

Colistin sulfate (C4461) and polymyxin B sulfate (5291) were purchased from Sigma-Aldrich (St. Louis, MO, USA). Colistimethate was manufactured by Sagent Pharmaceuticals (Schaumburg, IL) and ordered from the UT Southwestern Medical Center Pharmacy. *mcr-1*-positive *E. coli* isolates AF23, AF24, AF31, and AF45 were a generous gift from Patrice Nordmann (University of Fribourg, Fribourg, Switzerland) and were isolated from South African patients (12). The *mcr-1*-positive *E. coli* isolate MRSN 388634 was a generous gift from the Multidrug-Resistant Organism Repository and Surveillance Network (Walter Reed Army Institute of Research, Silver Spring, MD) and was the first reported *mcr-1*-positive clinical isolate in the United States (13). *E. coli* ATCC 25922 was obtained from the American Type Culture Collection (Manassas, VA).

### MIC and MBC assays. (i) Essential gene targets (**acpP** and **murA**).

MICs were determined with a modified Clinical and Laboratory Standards Institute (CLSI) MIC assay. Briefly, PPMOs were serially diluted (2-fold) in cation-adjusted Mueller-Hinton II broth (MHII). Overnight MHII cultures were diluted in fresh MHII to a concentration of 1 × 10^6^ CFU/ml and added 1:1 to diluted PPMO for a final concentration of 5 × 10^5^ CFU/ml, which was confirmed by serial dilution and plating on blood agar. Plates were incubated at 37°C for 18 h at 220 rpm, and then the optical density at 600 nm (OD_600_) was measured spectrophotometrically. The MIC was recorded as the lowest concentration with an OD_600_ of ≤0.06. MIC assays were conducted at least in triplicate, and if the MICs differed between replicates, the median was used. The growth curves to demonstrate that MCR-1 PPMOs and the Ctrl PPMO had no inhibitory activity were determined with 16 µM PPMO and incubated for 18 h, with OD_600_ readings taken every 15 min.

### (ii) Restoration of polymyxin activity.

The assay to determine the restoration of polymyxin activity was conducted as described above except that the antibiotic was serially diluted in MHII and then PPMO (Mcr1-0545, Mcr1-0638, or Ctrl-0949) was added to the bacterial suspension at a final concentration of 16 µM. Plates were incubated and MICs measured as described above.

### (iii) MBC assays for restoration of polymyxin activity.

MBC assays were conducted as described for the MIC assay except that aliquots were removed from respective wells at 18 h and diluted for CFU enumeration on blood agar.

### Mouse septicemia model.

Animal experiments were approved by the Institutional Review Board at UT Southwestern Medical Center (protocol number 2016-101626). The mouse model of septicemia with PPMO rescue of antibiotic activity was described previously (10).

### (i) Acute model.

Briefly, 7- to 8-week-old female BALB/c mice (Jackson Laboratory, Sacramento, CA, USA) were infected intraperitoneally (i.p.) with 8 × 10^4^ CFU of *E. coli* (MRSN 388634) in DPBS with 5% mucin (M1778; Sigma-Aldrich). At 2 and 6 h postinfection, 0.25 mg/kg colistin sulfate, 100 µg PPMO, or the combination was administered i.p. in 100 µl DPBS. Morbidity was assessed at 24 h, immediately prior to euthanasia. Mice were scored from 0 to 6 as follows: 0 for the absence or 1 for the presence of a ruffled coat; 0 for a normal posture, 1 for a hunched posture, and 2 for a prostrate posture; and 0 for nondisturbed movement, 1 for slow movement, 2 for movement after prodding, and 3 for no movement. Mice that succumbed to infection prior to euthanasia were scored as the maxima, 6 (moribund). Spleen and liver were harvested and homogenized in 1 ml DPBS (Omni TH; Omni International, Kennesaw, GA), serially diluted, and plated on blood agar.

### (ii) Survival model.

Mice were infected and treated as described above except that the inoculum was increased to 1 × 10^6^ CFU and the colistin dose was increased to 0.5 mg/kg. Mice were weighed, and morbidity was assessed every 6 h for the first 36 h and then every 12 h out to 120 h (5 days). Mice that reached the moribund state were euthanized.
