# Supplementary figures and images for: MCR-1 Inhibition with Peptide-Conjugated Phosphorodiamidate Morpholino Oligomers Restores Sensitivity to Polymyxin in Escherichia coli
Source: mBio. 2017 Nov 7;8(6):e01315-17. doi: 10.1128/mBio.01315-17 (PMC5676038; doi:10.1128/mBio.01315-17)

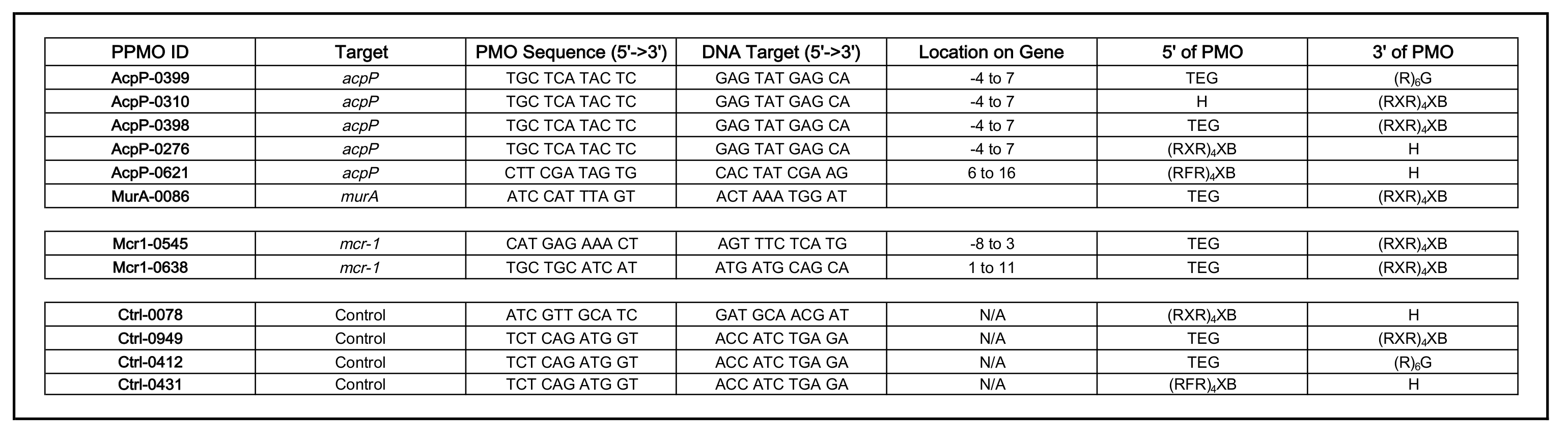

Supplement: TABLE S1 [file mbo005173574st1.tif]

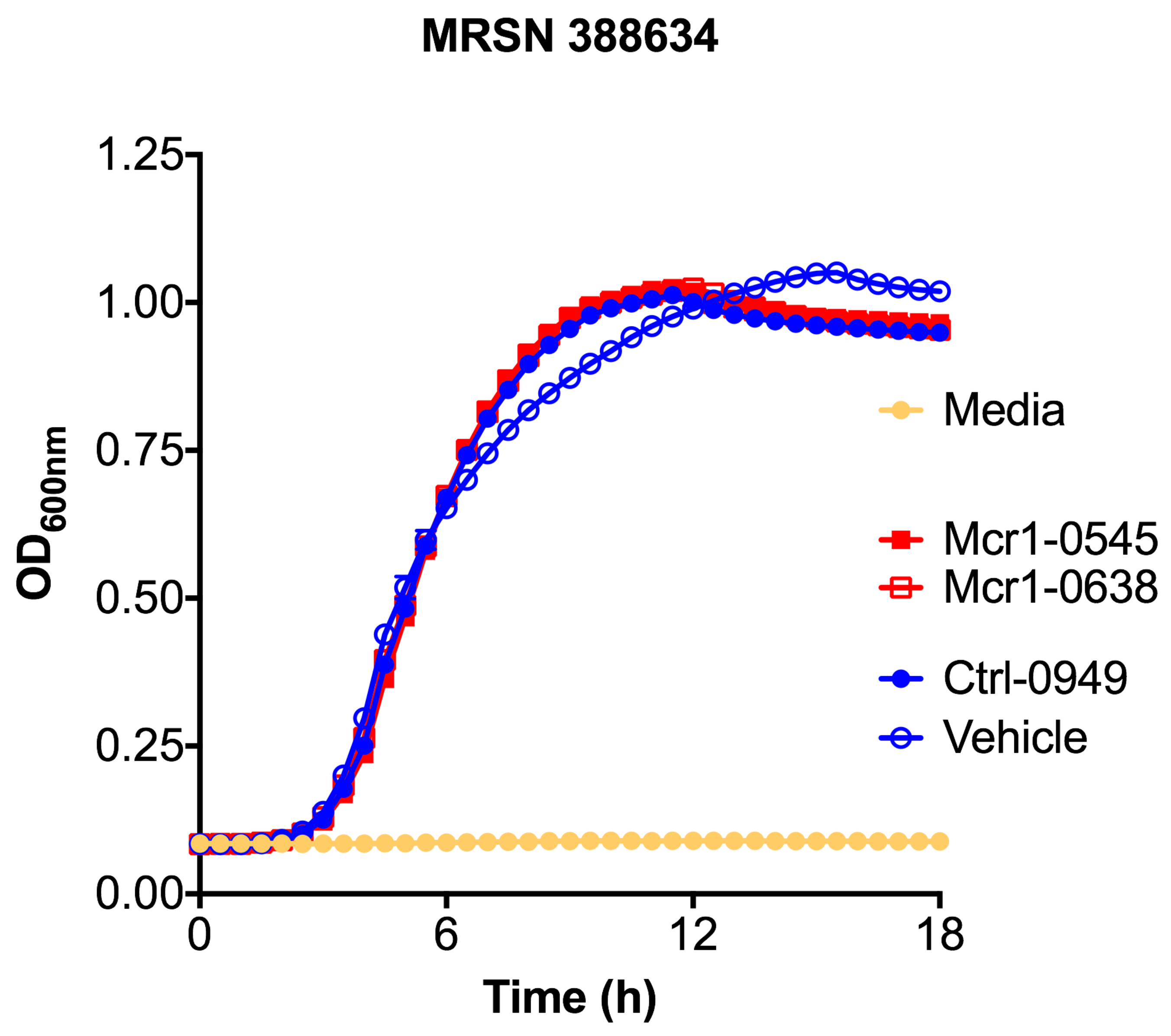

Supplement: FIG S1 [file mbo005173574sf1.tif]
