# Supplementary material for: MCR-1 Inhibition with Peptide-Conjugated Phosphorodiamidate Morpholino Oligomers Restores Sensitivity to Polymyxin in Escherichia coli
Source: mBio. 2017 Nov 7;8(6):e01315-17. doi: 10.1128/mBio.01315-17 (PMC5676038; doi:10.1128/mBio.01315-17)
Supplement: FIG S2 [file mbo005173574sf2.docx]

**Supplementary Figure 2**

**A**

**-20 to 20 of *mcr* genes**

Mcr1-0545 TCAAAGAGTAC
Mcr1-0638 TACTACGTCGT
**mcr-1 TATTTTTTGAGTAGTTTCTCATGATGCAGCATACTTCTGT**
mcr-1.2 TATTTTTTGAGTAGTTTCTCATGATGC**T**GCATACTTCTGT
mcr-1.3 TATTTTTTGAGTAGTTTCTCATGATGCAGCATACTTCTGT
mcr-1.4 TATTTTTTGAGTAGTTTCTCATGATGCAGCATACTTCTGT
mcr-1.5 T**G**TTTTTTGAGTAGTTTCTCATGATGCAGCATACTTCTGT
mcr-1.6 TATTTTTT**T**A**A**TAGTTTCTCATGATGCAGCATACTTCTGT
mcr-1.7 TATTTTTTGAGTAGTTTCTCATGATGCAGCATACTTCTGT
mcr-1.8 TATTTTTTGAGTAGTTTCTCATGATGC**G**GCATACTTCTGT
 * ****** * **************** ************

**mcr-1 TATTTTTTGAGTAGTTTCTCATGATGCAGCATACTTCTGT**
mcr-2 GGCATTTGTGGGTAATTTCTATGACATCACATCACTCTTG
mcr-3 GGACTATTAATGGAGTAAGTATGCCTTCCCTTATAAAAAT
mcr-4 TTAGCAAAAAAGGGGTTTTTGTGATTTCTAGATTTAAGAC
 * **

**B**

mcr-1 PMID: 26603172, KP347127
mcr-1.2 PMID: 27401575, KX236309
mcr-1.3 PMID: 28242671, KU934208
mcr-1.4 None, KY463451
mcr-1.5 PMID: 28678874, KY471308
mcr-1.6 PMID: 28264851, KY352406
mcr-1.7 None, KY463451
mcr-1.8 None, KY792081

mcr-2 PMID: 27416987, LT598652
mcr-3 PMID: 28655818, KY924928
mcr-4* PMID: 28797329, MF543359
 *Protein displays start as Met, but nucleotide start is Val
